# Tissue-Resident and Recruited Macrophages in Primary Tumor and Metastatic Microenvironments: Potential Targets in Cancer Therapy

**DOI:** 10.3390/cells10040960

**Published:** 2021-04-20

**Authors:** Tiziana Cotechini, Aline Atallah, Arielle Grossman

**Affiliations:** Department of Biomedical and Molecular Sciences, Queen’s University, Kingston, ON K7L 3N6, Canada; aline.atallah@queensu.ca (A.A.); arielle.grossman@queensu.ca (A.G.)

**Keywords:** tumor-associated macrophages, tissue-resident macrophages, tumor microenvironment, monocyte, metastasis-associated macrophage, trained immunity, depletion, recruitment, repolarization, cancer, immune therapy

## Abstract

Macrophages within solid tumors and metastatic sites are heterogenous populations with different developmental origins and substantially contribute to tumor progression. A number of tumor-promoting phenotypes associated with both tumor- and metastasis-associated macrophages are similar to innate programs of embryonic-derived tissue-resident macrophages. In contrast to recruited macrophages originating from marrow precursors, tissue-resident macrophages are seeded before birth and function to coordinate tissue remodeling and maintain tissue integrity and homeostasis. Both recruited and tissue-resident macrophage populations contribute to tumor growth and metastasis and are important mediators of resistance to chemotherapy, radiation therapy, and immune checkpoint blockade. Thus, targeting various macrophage populations and their tumor-promoting phenotypes holds therapeutic promise. Here, we discuss various macrophage populations as regulators of tumor progression, immunity, and immunotherapy. We provide an overview of macrophage targeting strategies, including therapeutics designed to induce macrophage depletion, impair recruitment, and induce repolarization. We also provide a perspective on the therapeutic potential for macrophage-specific acquisition of trained immunity as an anti-cancer agent and discuss the therapeutic potential of exploiting macrophages and their traits to reduce tumor burden.

## 1. Introduction

The tumor microenvironment (TME) is a diverse niche in which tumor, stromal, and immune cells dynamically interact via secreted factors and physical engagement, all within a dynamic extracellular matrix (ECM) [1]. Though the composition of the TME varies between tumor types, macrophages are often abundant and play a key role in tumor progression through promotion of tumor survival pathways and suppression of cytotoxic T cell responses [2]. Perhaps unsurprisingly, tumor cells co-opt a number of known tissue regulatory programs orchestrated by macrophage populations, including regulation of ECM, cell motility, chemotaxis, angiogenesis, and immune signaling pathways, to increase their survival [3]. Targeting macrophages and their tumor promotional programs is a major area of research in the pursuit of successful therapy [4]. It is also increasingly clear that targeting macrophages may be a beneficial approach to enhance the efficacy of conventional cytotoxic therapies and immune checkpoint blockade (ICB) [5]. Encouraging results from pre-clinical murine models are now being translated to the clinic with a number of clinical trials currently under investigation [6]. Here, we will discuss various macrophage populations, including tissue-resident as well as tumor- and metastasis-associated macrophages, as regulators of tumor progression and response to therapy. We will also evaluate the benefits of macrophage-targeted therapy and provide a perspective on macrophage-mediated targeted delivery of anti-cancer therapeutics.

## 2. Macrophage Nomenclature and Identification

Mills and colleagues first introduced the M1 and M2 paradigm of macrophage polarization as a corollary of Th1/Th2 polarization in the early 2000s [7]. This came about with the observation that macrophages from Th1-dominant mouse strains produced more nitric oxide (NO) in response to interferon-γ (IFNγ) or lipopolysaccharide (LPS) stimulation compared to macrophages from Th2-dominant strains stimulated with the same factors. In addition, the observation that macrophages from Th2-dominant strains could undergo arginase metabolism in response to LPS when compared with macrophages from Th1 counterparts dichotomized these two polarization states of macrophages [7]. In time, and with advances in genomics and transcriptomics, we have come to appreciate that the discreteness of these two populations is an oversimplification of complex, dynamic populations and could benefit from refinement [8,9]. Qian and Pollard have since argued that macrophage populations should be defined according to their functional and biological capabilities [8]. While there have been attempts to standardize macrophage nomenclature based on “the source of macrophages, definition of the activators, and a consensus collection of markers to describe macrophage activation”, a unifying language has yet to be accepted [10].

In the canonical sense, M1 macrophages are derived under the influence of IFNγ or LPS and secrete high levels of tumor necrosis factor-alpha (TNFα), interleukin (IL)-6, IL-12, and reactive oxygen species to promote inflammation and defend against pathogens [11]. Conversely, M2 macrophages develop in response to IL-4 and IL-13 and secrete high levels of IL-10 and transforming growth factor-β (TGFβ) to promote tissue repair and angiogenesis; they also modulate effector functions of lymphocytes to resolve inflammation [12]. Importantly, though the M1/M2 construct of macrophage polarization mirrors Th1/Th2 definitions, Th1/Th2 cells do not exclusively instruct macrophage polarization. Rather, macrophages orchestrate T cell polarization and effector responses, and thus, a clear understanding of these macrophage-mediated T cell modulatory processes is critical for understanding cancer progression. Despite some limitations, the M1/M2 paradigm has provided a useful, albeit simplistic, framework to classify macrophage-mediated processes involved in cancer progression. Whereas tumor promotional programs of macrophages in cancer have largely been described as M2-like within a Th2-skewed TME [13], anti-tumor macrophage properties are conversely attributed to M1 phenotypes and Th1-polarized responses [14]. Importantly, macrophages within the TME exhibit substantial plasticity and can alter polarization states when exposed to appropriate environmental cues [15]. Indeed, a major goal of macrophage-focused immune therapy is to induce repolarization of tumor-promoting macrophages to tumor-suppressive macrophages [16].

## 3. Monopoiesis and Macrophage Ontology

Our knowledge of monopoiesis and macrophage ontology largely comes from mouse studies due to a lack of methods and tools to evaluate human macrophage ontology [17] and the inability to study these early events during human development. Studies in mice revealed that macrophages have two developmental origins and can either be derived from circulating monocyte populations or from embryonic precursors [18]. In both mice and humans, two populations of circulating monocytes have been extensively characterized as classical monocytes (Ly6C^hi^ CCR2^+^ monocytes in mice; CD14^+^ CD16^−^ in humans) and non-classical/patrolling monocytes (Ly6C^lo^ CCR2^−^ in mice; CD14^lo^ CD16^+^ in humans) [19,20]. Whereas patrolling monocytes are primarily involved in vasculature maintenance [21], tissue recruitment of circulating classical monocytes occurs in response to injury, infection, inflammation, or neoplastic transformation [22]. This recruitment and differentiation of classical monocytes (Ly6C^+^ monocytes in mice or CD14^+^CD16^−^ monocytes in humans) is via CCR2-dependent pathways [23].

The majority of tissue-resident macrophage populations derive from both intra- and extra-embryonic precursors and are seeded in target organs before birth [18]. At the turn of the century, it was proposed that yolk-sac-derived phagocytes were a separate developmental lineage from hematopoietic precursors [21]. Indeed, in the mouse, macrophages arise from two distinct waves of hematopoiesis and two distinct lineages of cells with differential dependence on the transcription factor c-myb. In the first c-myb-independent wave, yolk-sac blood islands give rise to colony-stimulating factor-1 receptor positive (CSF-1R^+^) erythromyeloid progenitors (EMPs) on embryonic day (ED) 7.5, prior to initiation of blood circulation. The CSF-1R is encoded by the proto-oncogene *c-fms* [24] and is a major lineage regulator for the majority of macrophage populations [25]. By ED 8.5, EMPs differentiate into primitive macrophages expressing the lineage markers F4/80^+^ and CX3CR1^+^ [26] and begin to seed organs in the embryo [18]. Though fate-mapping studies have provided evidence that yolk-sac derived primitive macrophages give rise to tissue-resident cells in the lung, liver, gut, pancreas, and skin [24], only skin resident Langerhans cells and brain resident microglia retain this ontology, Ref. [27] whereas other tissue-resident cells derive from hematopoietic stem cells (HSCs), which originate during the second wave of hematopoiesis [28].

During the second wave of fetal monopoiesis, which is c-myb dependent, yolk-sac EMPs migrate to the fetal liver by ED 9.5 and give rise to definitive HSCs, including CSF-1R^+^ myeloid progenitors [18]. These fetal monocytes then travel to the viscera via the circulation and differentiate into definitive macrophages as they enter the organs, replacing the majority of previously-seeded yolk-sac derived primitive macrophages. With the exception of intestinal macrophages, which are primarily maintained by circulating monocytes [29], the majority of these fetal-derived resident macrophage populations, including brain microglia, liver Kupffer cells, alveolar macrophages, and splenic red pulp macrophages [30], are largely maintained by self-renewal under steady-state. However, experimental evidence has shown that bone-marrow-derived circulating monocytes can give rise to self-renewing tissue-resident populations if an appropriate niche is available. For example, Scott and colleagues reported the ability of circulating monocytes to differentiate into liver-resident Kupffer cells following diphtheria-toxin-mediated depletion of the endogenous Kupffer cell population [31]. Moreover, alveolar macrophages, cardiac macrophages, F4/80^+^ brain microglia and barrier-associated macrophages in the brain can be replenished by circulating monocytes following age- or inflammation-associated cellular loss [28,32,33]. However, it is important to note that, in most cases, the recruitment of bone-marrow-derived cells does not entirely replace tissue-resident macrophages [34]; thus, the contribution of both recruited and tissue-resident macrophage populations are important considerations when studying disease states. Another important consideration arises from intriguing findings of a recent fate-mapping study, which revealed that macrophage homeostasis is achieved in mouse tissues by 12–20 weeks of age. This suggests that macrophages in 6–8 week old mice, which are primarily utilized in preclinical studies, may not be fully mature [34].

## 4. Tissue-Resident Macrophages

Tissue-resident macrophages reside in the majority of adult organs and include lung alveolar macrophages; epidermal Langerhans cells; dermal macrophages; liver Kupffer cells; splenic red pulp macrophages; brain microglia; bone osteoclasts; large peritoneal macrophages; F4/80^bright^ pancreatic macrophages; and kidney, cardiac, adipose tissue, and mammary gland macrophages [18,34,35]. With the advent of more sophisticated fate mapping technologies, additional populations of tissue-resident macrophages with embryonic origin are likely to be discovered. Indeed, new evidence has shown that lung interstitial macrophages derive from the embryo and can be replenished by circulating bone-marrow-derived cells [36]. Moreover, using a fate-mapping approach, De Schepper and colleagues challenged the dogma that all intestinal macrophages are continuously replaced by bone-marrow-derived Ly6C^+^ monocytes [37]. Their work revealed that embryonic-derived, self-maintaining gut macrophages colonize and remain in anatomically distinct intestinal niches, including the vasculature, submucosal and myenteric plexus, and Peyer’s patches, into adult life.

Tissue-resident macrophage populations are a heterogenous population of cells that are less plastic than their recruited counterparts [38] and exhibit substantial tropism relative to their microenvironment. Genomic studies have revealed that there is significant genetic diversity among tissue-resident macrophage populations [39] despite sharing a common developmental origin. As described above, tissue-resident macrophages are the first leukocyte lineage to develop during embryogenesis [18,29]. Thus, these resident cells are critical to maintaining tissue homeostasis and controlling tissue remodeling and organ development [40]. For example, resident macrophage populations in nulliparous mammary glands are key to maintenance of ECM homeostasis [41]. Interestingly, recent work has definitively shown that these fetal-derived macrophages populate the mammary gland before birth and outnumber recruited macrophages into adulthood [42], suggesting that preservation of their tissue remodeling phenotype is important throughout life.

Critical roles of tissue-resident macrophages and their dependence on CSF-1R signaling were elucidated with the discovery and use of two key mouse models with disruptions in the CSF-1/CSF-1R signaling axis. Examination of tissues from *Csf1*^op/op^ mice [43], which have a naturally occurring deletion in the CSF-1 gene, and *Csf1r*^-/-^, which have a targeted null deletion in *csf1r* [44], have underscored the importance of the CSF-1/CSF-1R signaling axis in macrophage survival, proliferation, and differentiation. *Csf1*^op/op^ mice lack tissue-resident macrophages, are osteoclast deficient, have impaired pancreatic cell proliferation (including reductions in insulin-producing beta cells [45]), and show defects in mammary pad ductal branching both postnatally [46] and during pregnancy [47]. Defects observed in *Csf1r*^-/*-*^ mice phenocopy *Csf1*^op/op^ mice but are more severe, thus, emphasizing the role of CSF-1R signaling by CSF-1-independent factors in macrophage development and survival [44]. Indeed, Lin and colleagues identified IL-34 as a second binding partner for CSF-1R [48]. Though both ligands similarly induce monocyte to macrophage differentiation, CSF-1R engagement by each ligand activates differential signaling pathways and distinct cytokine profiles from polarized macrophages [49,50]. For example, IL-10 released from IL-34 M1-polarized macrophages was found to be increased compared to the amount of IL-10 released from CSF-1 M1-polarized macrophages [50]. Nevertheless, targeting the CSF-1/CSF-1R axis using small molecule inhibitors or neutralizing antibodies depletes many tissue-resident populations given their dependence on this signaling axis for survival. Alveolar macrophages are a notable exception; in adult life, this resident population is dependent upon GM-CSF signaling for maintenance and survival [51,52].

Tissue-resident macrophages are also important antigen-presenting cells positioned to act as sentinels of the immune system and, thus, play important roles in controlling infection and resolving inflammation [53]. These resident populations are anatomically well-positioned to sample air- or blood-borne antigens through phagocytosis and expression of receptors that recognize pathogen-associated molecular patterns (PAMPs) and damage-associated molecular patterns (DAMPs) [54]. Following antigen processing, macrophages present and cross-present to CD4^+^ and CD8^+^ T cells [55]. In addition, there is evidence that selective ablation of resident macrophages impairs neutrophil infiltration in response to infection, thus, highlighting the role of tissue-resident macrophages in the initiation of immune responses [55]. Resident macrophages also coordinate and maintain tolerogenic states in the organs in which they reside. This can be intrinsically maintained by tolerogenic transcriptional programs [56], expression of immune checkpoint molecules (i.e., programmed-death ligand (PD-L)-1 and PD-L2) [57], and generation of FoxP3^+^ regulatory T cells [57]. For example, alveolar macrophages abundantly express PD-L1, resulting in dampened immune responses [58], and have also been shown to restrict dendritic cell presence in airways [59]. This leads to attenuated antigen presentation capacity of local dendritic cells and impairs dendritic cell-mediated T cell proliferation [60]. These steady-state characteristics of alveolar macrophages and other resident immune macrophages are reminiscent of the immune-modulating, tumor-promoting functions of tumor-associated macrophages (TAMs) and may represent targets for improving anti-cancer therapy.

### 4.1. Tissue-Resident Macrophages in Cancer

During neoplastic development or seeding of metastatic tumor cells, tissue-resident macrophages are among the first cells to interact with transformed cells and, thus, are likely to play an important role in tumorigenesis. However, relatively little is known about the role of tissue-resident macrophages in controlling tumor promotion and progression. This is in part due to the fact that we have only recently been able to understand the origin and ontology of tissue-resident macrophage populations using modern and sophisticated fate-mapping tools combined with genetically engineered mouse models [18,29].

A hallmark of the TME is Th2-driven inflammation [61], and evidence has shown that IL-4, a canonical Th2 cytokine, drives tissue-resident macrophage proliferation and expansion [62]. Thus, in the background of cancer, the tumor-promotional phenotypes of tissue-resident macrophages may be amplified. Macrophages can also promote different aspects of carcinogenesis depending on their developmental origin. In the mouse lung, where monocyte-derived macrophages were found to promote tumor metastasis, tissue-resident macrophages were found to correlate with tumor growth [63]. These results are corroborated by an additional study, which found that intratracheal L-Clodronate-mediated depletion of resident alveolar macrophages did not impact experimental metastasis of mammary carcinoma-derived Met-1 cells [64]. On the contrary, in a mouse model of pancreatic ductal adenocarcinoma (PDAC), selective depletion of resident macrophages reduced tumor progression when compared with deletion of recruited macrophage populations [65]. Together, these data suggest that both macrophage origin and the specificity of tissue residency may differentially impact tumor progression.

Tissue-resident and recruited macrophage populations reveal differences in repopulation following therapeutic interventions or depletion strategies. For example, monocyte-derived TAMs recovered faster than resident macrophages in response to chemotherapy in a mouse model of lung cancer [63]. Thus, the contribution of each macrophage lineage should be an important consideration when designing drug combination trials. As expected, under steady-state conditions, antibody-mediated targeting of CSF-1R reduces maturation presence of tissue-resident macrophage populations while having no effect on the population of Ly6C^+^ inflammatory monocytes [66]. However, in syngeneic tumor models, anti-CSF-1R treatment also depleted TAMs, suggesting either similarities between tissue-resident macrophages and TAM populations or a common cellular origin of these two populations [66,67]. Indeed, both monocyte-derived and tissue-resident macrophages were identified as TAMs in mouse models of PDAC [65], glioblastoma (GBM) [67], and lung cancer [68]. In the PDAC model, self-renewing fetal-derived tissue-resident macrophages exhibited a distinct pro-fibrotic transcriptional profile compared with monocyte-derived TAMs. Moreover, while selective depletion of TAMs did not impact PDAC growth, depletion of tumor-resident macrophages significantly inhibited tumor progression [65]. In contrast, Atunes and colleagues recently reported that whereas resident microglia-derived TAMs predominate during early GBM diagnoses, monocyte-derived macrophages are associated with recurrence [67]. Thus, the relative contribution of developmentally distinct macrophage populations on cancer progression may be tissue-specific, and thus, a more complete understanding of macrophage ontology in cancer is needed.

### 4.2. Tissue-Resident Macrophages in Metastasis

Subsets of both monocyte-derived and tissue-resident macrophages contribute to metastasis in various ways and have been termed metastasis-associated macrophages (MAMs) [69]. Tumor cells exhibit tissue tropism for metastatic spread to distant sites; however, it is unknown whether this is associated with intrinsic differences in tissue biology or if it arises as a function of different properties and phenotypes of resident immune cell populations in these sites. When viewed in the context of the M1/M2 paradigm, tissue-resident macrophages are generally ‘M2-like’, owing to their fundamental roles in coordinating tissue development, maintaining tissue integrity, iron homeostasis, and resolving inflammation [70]. These cells may therefore contribute to establishment of the pre-metastatic niche by maintaining an optimal ‘soil’ for the eventual seeding of disseminated tumor cells. The use of experimental metastasis models reveals a propensity for circulating tumor cells to seed in the lung, liver, brain, and bone. Unsurprisingly, these sites are populated by resident tissue macrophages, including alveolar macrophages, Kupffer cells, microglia, and osteoclasts, respectively. While anatomical considerations are often used to explain the preferential seeding of tumor cells at these sites, functional properties of the local tissue microenvironment, including the tumor-promotional properties of tissue-resident macrophages at these sites, cannot be overlooked. Indeed, CD163^+^ TIM4^+^ embryonically-derived resident omental macrophages were found to contribute to ovarian cancer metastasis by supporting acquisition of epithelial–mesenchymal transition characteristics and stem cell-like phenotypes in ID8 cells [71]. Targeted depletion of this macrophage population using genetic targeting or antibody-targeted cytotoxic liposomes prevented metastatic disease in this model. In conjunction with the findings by Zhu and colleagues described above [65], these data support the notion that therapeutic targeting of resident macrophage populations may be necessary to prevent malignant progression.

## 5. Tumor-Associated Macrophages

Tumor-associated macrophages are largely recruited to the TME from circulating monocytes derived from hematopoietic progenitors in bone marrow [72]; although, as discussed above, some TAMs may in fact be descendants of tissue-resident cells populated by self-renewal [14]. Regardless of their origin, cross-talk between tumor cells and macrophages within the TME drives cancer progression. Release of CSF-1 from tumor cells promotes recruitment and survival of macrophages within the TME, and these cells, in turn, promote tumor cell migration via release of macrophage-derived epidermal growth factor [73]. Cancer cells can also influence the polarization of recruited macrophages through release of soluble factors. For example, lung cancer cells were found to promote M2 polarization through the release of IL-37, leading to macrophage expression of MARCO (macrophage receptor with collagenous structure) and the induction of tumor-promoting phenotypes [74].

In addition to promoting the acquisition of malignant phenotypes, TAMs are an important component of the tumor microenvironment of metastasis (TMEM). Using intravital microscopy, Harney and colleagues described the TMEM—a unique niche consisting of a macrophage, tumor cell, and endothelial cell—as a site of tumor cell intravasation [75]. Within the primary tumor TMEM, vascular endothelial growth factor (VEGF)-A signaling from Tie2^hi^ macrophages facilitates increased vascular permeability and coordination of tumor cell intravasation leading to distant spread [75]. More recently, macrophages and, in particular, macrophage-derived Wnt ligands, were shown to orchestrate early dissemination of pre-malignant tumor cells in a mouse model of HER2+ mammary cancer [76]. Intriguingly, CSF-1R-mediated macrophage depletion during the pre-malignant phase in the absence of a palpable tumor significantly reduced lung metastatic burden [76]. Remarkably, this reduction in disease burden was conserved after one month of therapy cessation, thus, enforcing the role of macrophages within the TMEM as critical drivers of early dissemination and metastasis [76]. Together, this work provides a rationale for disruption of the TMEM as a critical checkpoint for preventing metastatic spread.

### Prognostic Significance of TAM Density across Different Tumor Types

Despite significant advances in our understanding of the role of the tumor immune microenvironment in cancer progression, clinical assessment of cancer still relies on intrinsic tumor parameters as defined by the TMN classification system [77]. The introduction of the Immunoscore, pioneered by Jerome Galon, has proven to have significant predictive and prognostic value, surpassing that of the TMN system in colorectal cancer. The utility of this quantitative scoring system, which evaluates the presence of CD3^+^ and CD8^+^ T cells at the tumor center and invasive margin, has the potential to be the first clinical tool to evaluate tumor progression as a function of immune contexture [78]. No such ‘macrophage immunoscore’ has been as extensively studied in human cancers, likely owing to difficulties associated with accurate detection and phenotyping of macrophage populations using traditional immunohistochemistry approaches [79]. Although often used to identify macrophages, CD68 as a single marker is not ideal since it does not exclusively detect macrophages [80] and cannot adequately stratify polarization states of macrophage populations. For robust detection and immunolocalization, a combination of markers is needed, including but not limited to HLA-DR, CSF-1R, CD163, CD68, and CD206 [79]. A number of pre-clinical multiplexing approaches have been designed to facilitate immunodetection of multiple proteins on a single slide [81,82,83], though each comes with barriers to accessibility in the clinic, including lack of validation, high cost, and inefficiency.

Despite a lack of robust TAM markers, results from both preclinical mouse models and human studies have revealed that macrophages are a crucial component of the TME and contribute to cancer progression through a variety of mechanisms, including modulation of the adaptive immune system. Indeed, the CD68^+^ to CD8^+^ ratio in breast [84] and pancreas [85] tumors has been shown to predict survival such that patients with CD68^hi^ CD8^lo^ tumors succumb to their disease faster than patients with CD68^lo^ CD8^hi^ leukocyte density. Similar findings in hepatitis B virus-related hepatocellular carcinoma were recently described. In this work, the authors examined matched tumor and paratumoral stroma and found that a high CD68^+^ to CD8^+^ ratio in both regions was correlated with reduced overall survival [86].

In endometrial cancers, high TAM density, and in particular, density of CD163^+^ TAMs, a marker of M2 and tissue-resident macrophages, correlated with poor progression-free survival [87]. A recently published prospective study in ovarian cancer revealed the prognostic role for M1-polarized TAMs in high-grade serous carcinoma [88], where patients with high M1/M2 ratios had longer overall and progression-free survival. The best outcomes were observed in patients with higher M1 TAM levels who also underwent surgery and chemotherapy, suggesting that therapeutic opportunities combining standard of care treatment with macrophage repolarization strategies may be important in achieving tumor control. Indeed, a recent study of patients with stage II colon cancer found that adjuvant chemotherapy improved survival in patients with a high CD206/CD68 ratio of TAMs, indicative of an M2-skewed TME [89]. For this disease, and a number of others where adjuvant chemotherapy generally has no effect on prolonging survival, the use of a simple and inexpensive predictive biomarker to stratify patients based on therapeutic success is paramount to alleviating unnecessary patient suffering and reducing adverse events [89].

One of the largest meta-analyses investigating the prognostic value of TAMs across multiple tumor types revealed that TAM density is associated with worse prognosis in head and neck cancer, gastric cancer, breast cancer, bladder cancer, ovarian cancer, thyroid cancer, urogenital cancer, and better overall survival in colorectal cancer [90]. Interestingly, this study found no observable effect of M1/M2 TAM phenotype on overall survival, which is contrary to the results of another meta-analysis, which found that M2 TAM polarization was predictive of poor survival in patients with gastric cancer [91]. In bladder cancer, high presence of CD68^+^ TAMs correlated with negative health outcomes, including a higher risk of necessitating cystectomy, vascular invasion, and distant metastasis and had lower overall survival (five year survival: 47% in high TAM group vs. 97% in low TAM group) [92]. Moreover, in that work, the authors found that the density of TAMs in muscle-invasive bladder cancer was higher than in cases of superficial bladder cancer, suggesting that TAM presence parallels malignant progression.

While many of the studies described above point to an inverse relationship between TAM density and prognosis, recent work evaluating morphological characteristics of macrophages in colorectal liver metastasis found cell size to be an indicator of TAM diversity that also tracked with prognosis [93]. In that study, the authors proposed that differences in cell area delineate resident versus recruited cells such that large macrophages are resident Kupffer cells whereas small macrophages are recruited TAMs. Interestingly, the presence of small macrophages in patient samples was associated with prolonged disease-free survival when compared with patient samples exhibiting a preponderance of large macrophages [93].

In addition to the predictive value of immunohistochemical localization and assessment of macrophage populations across solid tumors, many groups have described ‘TAM gene signatures’ as robust prognostic indicators of outcome [94,95,96]. A recent large-scale transcriptomic study comprised of publicly available human breast cancer data from more than 1000 patients found that, while a high M1 signature [97] correlated with a favorable TME, transcriptomically high M1 tumors were associated with clinically aggressive features and had no survival benefit [98]. A possible explanation for this surprising result includes the finding that, in this study, M1-skewed tumors were more proliferative given their association with higher Ki67 expression. In addition, M1-skewed tumors exhibited increased expression of inhibitory checkpoint molecules, including PD-L1, and T-cell exhaustion markers, including programmed death receptor-1 (PD-1), lymphocyte-activation gene-3 (LAG-3), and cytotoxic T-lymphocyte-associated protein-4 (CTLA-4) [98]. On the other hand, Cassetta and colleagues identified a 37-gene TAM signature expressed in highly-aggressive breast cancers associated with reduced disease-specific survival in a cohort of more than 1900 patients [95]. Additionally described in this work was the intriguing finding that TAMs isolated from human endometrial and breast cancers are transcriptionally distinct from both monocytes and their tissue-resident counterparts, once again highlighting the heterogeneity of macrophage populations [95].

The use of single-cell assessment techniques, including single-cell RNAseq, mass cytometry, imaging mass cytometry, and multiplexed imaging techniques, have greatly enhanced our understanding of unique and heterogenous cell populations within tumors. These techniques are being exploited to create extensive single-cell atlases of the immune contexture across various cancer types, including clear cell renal cell carcinoma [99], PDAC [100], and breast cancer [99,101]. Use of these techniques has revealed previously unknown information about macrophage ontology and specialization and has identified subsets of myeloid cells that are conserved between humans and mice [67]. Data from these experiments also highlight the notion that TAMs exhibit extensive phenotypic diversity such that categorization of these cells along the M1/M2 spectrum is difficult and problematic. For example, a mass cytometry study on renal cell carcinoma revealed 17 different TAM phenotypes [99]. This highlights the notion that canonical classifications and phenotypes of TAMs will likely require modification as we dive deeper into single-cell data. Nevertheless, these techniques provide insight into macrophage complexity and will be important for developing macrophage-targeted anti-cancer therapies.

## 6. Metastasis-Associated Macrophages (MAMs)

Macrophages are instrumental in all steps required for metastatic spread of tumor cells, including: (1) local invasion and migration; (2) intravasation; (3) survival in the circulation; (4) arrest at secondary site; (5) extravasation; and (6) survival and outgrowth [102]. As already discussed above, tumor-promotional programs of TAMs facilitate unchecked expansion of tumor cells, while TAMs present within TMEM facilitate intravasation. Once in the circulation, tumor cells associate with leukocytes, including neutrophils and monocytes. This union drives the expression of cell-cycle and metastasis-promoting genes, which ultimately expands the metastatic potential of circulating tumor cells [103]. Macrophages are then recruited to the site of extravasating tumor cells where they promote seeding, growth, and survival of malignant cells [64]. Moreover, tumor cell expression of vascular cell adhesion molecule-1 (VCAM-1), a leukocyte adhesion molecule canonically expressed on the endothelium, results in tethering of tumor cells to macrophages, and this cell-to-cell interaction promotes survival signals in metastatic cells [104]. Similarly, hybrid cells resulting from the fusion of macrophages with tumor cells exhibit greater motility, invasiveness, seeding, and outgrowth in metastatic sites compared with non-hybrid cells; moreover, the presence of circulating hybrids correlates with tumor stage and survival in human patients [105].

A series of elegant mouse studies from Jeffrey Pollard’s group has greatly contributed to our understanding of the biology of MAMs, in particular, how CCL2-mediated recruitment of CCR2^+^ tumor-promoting inflammatory monocytes facilitates metastasis [64,69,106,107]. Recruitment of classical Ly6C^+^ blood monocytes to tumor-challenged lungs can be inhibited in a CCR2-mediated manner, reducing MAMs and alleviating metastatic burden [108]. These results align with work from others revealing that CCR2-mediated depletion of MAMs restricts outgrowth after initial seeding of bone metastases in a mouse model of mammary carcinoma [109]. Moreover, in addition to promoting tumor cell extravasation via local release of VEGF-A [108], CCR2-expressing monocytes give rise to immune-suppressive MAM-precursors that exhibit resistance to CSF-1 blockade [69] and promote pulmonary seeding of mammary carcinoma cells [107].

TAMs, MAMs, and tissue-resident macrophages all express the VEGF-A receptor, VEFGR-1, though expression is higher in MAMs than in resident macrophages [64]. Targeting VEGFR1-expressing macrophages was found to reduce seeding efficiency and metastatic outgrowth in experimental and spontaneous mouse models of mammary carcinoma [106]. Moreover, loss of caveolin-1 expression in MAMs increased VEGFR-1 expression and promoted metastasis, which supports the pro-metastatic role for VEGFR-1-expressing MAMs [110]. Indeed, evaluation of patient samples revealed that the number of circulating pro-angiogenic VEGFR-1^+^ MAMs is predictive of liver metastasis in colorectal cancer [111].

## 7. Macrophages and Therapeutic Efficacy

Response to therapy in cancer depends on several tumor cell intrinsic and extrinsic factors, the latter including the TME [112]. In addition, macrophages dynamically respond to danger signals induced by ionizing radiation [113] and cytotoxic therapy [114]. It is not surprising, therefore, that the phenotype and presence of tissue-resident and recruited TAMs play a role in modulating responses to ICB and conventional therapies, including chemotherapy and radiation therapy [115,116,117].

### 7.1. Macrophages Restrict T Cell Mobilization and Anti-Tumor Immune Responses

Tissue-resident macrophages reside and persist in sites where maintaining tissue homeostasis and tempering inflammation is critical to maintenance of non-pathological states [118]. Intrinsic to many resident macrophage populations is the ability to restrict T cell activity in response to the tissue microenvironment [119,120,121]. This can be achieved through expression of inhibitory immune checkpoint molecules, including PD-L1 and PD-L2 [119], impaired antigen presentation [120], secretion of immunosuppressive cytokines (including IL-10, TGF-β, prostaglandin E_2_) [118], and depletion of tryptophan [122] and arginine [123], which are necessary for T cell fitness. In human hepatocellular carcinoma, PD-L1 expression on Kupffer cells in tumor-rich areas was higher than in adjacent normal tissues and correlated with poorer survival [124]. In the context of cancer, TAMs and MAMs adopt similar T cell suppressive phenotypes as resident macrophages, ultimately resulting in impaired anti-tumor immunity and responses to therapy.

Durable and effective tumor control is achieved by mobilization and activation of cytotoxic CD8^+^ T cell responses. As is the case for resident macrophages, TAMs and MAMs are directly associated with impaired activation of CD8^+^ T cells [125] and can restrict mobilization into [126,127] and migration within [127] solid tumors. TAMs, MAMs, and resident macrophages may also facilitate T cell exclusion in tumor nests via induction of collagen remodeling enzymes, including matrix metalloproteinases (MMPs) and cathepsins, and initiation of fibrosis [65,128,129]. Interestingly, the CD206 mannose receptor, which is also a marker for M2-like macrophages and TAMs, was found to induce T cell tolerance via inhibition of CD45 phosphatase activity and upregulation of CTLA-4, an inhibitory immune checkpoint molecule [130]. Selective depletion of TAMs results in increased presence of CD8^+^ T cells and enhances the response to chemotherapy [84,131,132,133], radiotherapy [134], and ICB [127,135]. Furthermore, a recent study describing T cell-induced release of CSF-1 as a CD8^+^ T cell-dependent adaptive resistance mechanism in melanoma highlights the importance of macrophage depletion in tumors refractory to ICB [136].

### 7.2. Macrophages Impair Responses to Chemotherapy and Radiotherapy

Induction of chemotherapy and radiation therapy causes the release of soluble factors, including VCAM-1 and CCL2 [137,138], which leads to robust macrophage infiltration to the TME. Since macrophages are known drivers of therapy resistance [139], this treatment-induced recruitment perpetuates the cycle of therapy resistance that culminates in relapse [117,137,140,141,142,143]. In vitro, prostate cancer cells treated with docetaxel released more CSF-1 and recruited more macrophages in a transwell assay compared with untreated cells. Subsequent in vivo work using castration-resistant prostate cancer models confirmed that combining the macrophage depleting agent PLX3397 with docetaxel improved chemotherapeutic response, resulting in durable tumor growth suppression [144]. In response to chemotherapy, Ruffell and colleagues demonstrated that macrophage-derived IL-10 limits effective cytotoxic CD8^+^ T cells via suppression of IL-12 expression from intratumoral dendritic cells in the MMTV-PyMT model of mammary carcinoma [145]. Macrophage-mediated IL-10 release has also been shown to activate the IL-10 receptor/STAT3/Bcl-2 pathway [146], leading to cancer cell survival [146,147]. Indeed, STAT3 is an important signaling molecule involved with cell survival and cell cycle activation processes, including caspase inactivation [148,149], which is a common mechanism of chemotherapy-induced resistance [150]. Activation of STAT3 also confers therapy resistance through other mechanisms, including activation of the transcription factor, nuclear factor kappa-light-chain-enhancer of activated B cells (NF-κB) [147,151]. Macrophage-derived TNFα and IL-6 induced therapy resistance in estrogen receptor positive (ER+) breast cancer cells following activation of NF-κB pathways [151]. In particular, downstream ERK activation led to phosphorylation and constitutive expression of ER, resulting in sustained proliferation [151].

Phenotypic classification of therapy-induced infiltrating macrophage subsets may provide insight into their origin and contribution to resistance [117,138,143] in a tissue-specific or tumor-specific manner. For example, CX3CR1^+^ macrophages increase following radiotherapy and mediate resistance in a mouse model of lung cancer [143]. On the other hand, Ly6C^+^ CCR2^+^ CX3CR1^lo^ inflammatory monocytes have been found to infiltrate tumors following radiotherapy, wherein they promote resistance [138]. Tissue-resident macrophage populations are generally CX3CR1^hi^, compared with CX3CR1^lo^ cells found in bone marrow [152]. Thus, an appreciation for macrophage ontology may be beneficial to understanding and overcoming therapy resistance.

Cancer cell/TAM cross-talk promotes malignant progression [153] and these interactions often occur in response to therapy. For example, a recent in vitro study found that cisplatin-induced release of CCL20 from classically activated macrophages promoted ovarian cancer cell and cell migration via the CCL20–CCR6 axis [154]. Moreover, macrophages are important mediators of soluble factor mediated drug resistance [155]. Resistance to 5-Fluorouracil, which occurs in a large proportion of patients with colorectal cancer, is mediated in part by chemotherapy-induced activation of macrophages and subsequent release of protective factors, such as putrescine, to inhibit tumor cell apoptosis [156]. Moreover, following cisplatin chemotherapy, macrophage-mediated exosomal delivery of microRNA-21 (miRNA-21) to gastric cancer cells was found to suppress tumor cell apoptosis, thus, promoting therapy resistance [157]. Indeed, expression of miRNA-21 is associated with poor survival and poor response to chemotherapy in multiple cancers, including ovarian and colon cancer [158,159].

### 7.3. Macrophages Impair Immune Checkpoint Blockade Responses

TAMs and resident macrophages robustly express inhibitory checkpoint molecules, including PD-L1, PD-L2, and PD-1, and thus can function to sequester neutralizing antibodies intended for T cell targets used in ICB therapy [6]. Moreover, the effectiveness of antibody-mediated checkpoint blockade therapy was found to depend on the antibody’s Fc domain. Using in vivo imaging techniques, Arlauckas and colleagues observed TAMs removing anti-PD-1 antibodies from the surface of T cells via their Fcγ receptors (FcγRs) but also found that blocking FcγRs prolonged the effects of checkpoint blockade [160]. Results from a number of tumor models have substantiated the finding that macrophages limit response to ICB such that the addition of macrophage depleting agents synergizes with ICB and leads to anti-tumor responses in refractory tumors [161]. Indirectly, macrophages can limit response to ICB via release of soluble factors that orchestrate tissue remodeling events to thereby restrict tumor access to CD8^+^ T cells. In a model of metastatic PDAC refractory to anti-PD-1 checkpoint blockade, inhibition of CSF-1-mediated release of granulin from TAMs prevented fibrosis and restored anti-tumor immune responses, leading to reduced metastatic burden [129].

## 8. Targeting Macrophages to Potentiate Anti-Cancer Therapy

**Figure 1:**
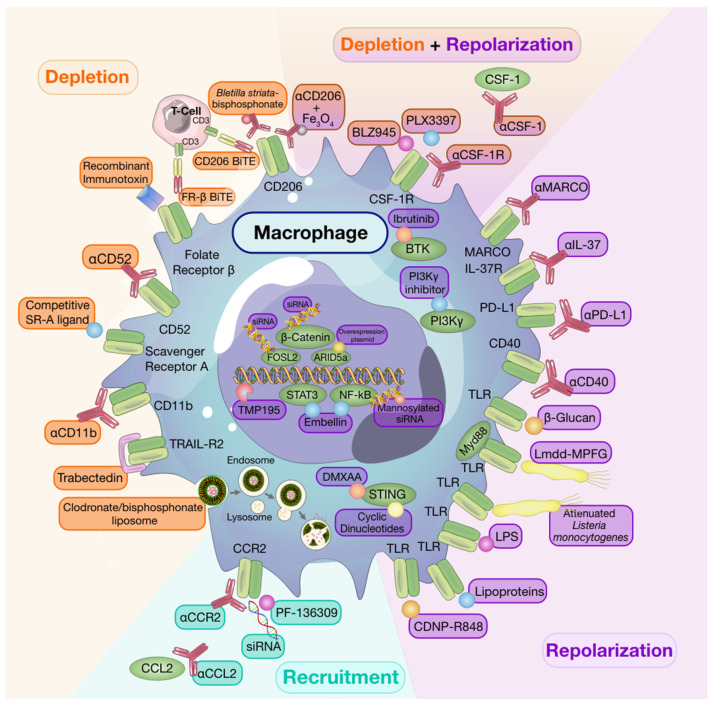
Therapeutic strategies targeting macrophages and their tumor-promoting phenotypes. Macrophage depletion, inhibiting macrophage recruitment, and/or promoting repolarization of macrophages towards anti-tumor phenotypes are the main strategies currently under investigation in the pursuit of developing macrophage-targeted therapies in cancer. These strategies include the use of antibodies (αCSF-1, αCSF-1R, αCCR2, αCCL2, αCD11b, αCD52, αCD206, αMARCO, αIL-37, αPD-L1, αCD40); small molecule inhibitors (PLX3397, BLZ945, embelin; ibrutinib); pharmacological inhibitors (PF-136309); recombinant immunotoxin; small-interfering RNAs; toll-like receptor (TLR) agonists (CDNP-R848; lipopolysaccharide [LPS]; lipoproteins); stimulator of interferon genes (STING) agonists (DMXAA; cyclic dinucleotides); trabectedin chemotherapy; clodronate or bisphosphonate-packed liposomes; microbe-derived products (β-glucan, attenuated listeria monocytogenes, attenuated hepatocellular carcinoma-specific Listeria vaccine [Lmdd-MPFG]); histone deacetylase inhibitors (TMP195); competitive inhibitors (competitive scavenger receptor A [SR-A] ligand); novel antibody-drug conjugates, such as bivalent T cell engagers (BiTE; folate receptor-β [FR-β] BiTE, CD206 BiTE); and antibody-conjugated iron oxide nanoparticles (αCD206 + Fe_3_O_4_) in the blockade of macrophage survival, recruitment, and repolarization pathways.

Given the high propensity of macrophages as prognostic indicators of negative outcomes in solid tumors, it stands to reason that selective and targeted therapeutic approaches should benefit the patient. Indeed, over the past two decades, results from pre-clinical studies have largely established a tumor-promotional role of macrophages in solid tumors [4,14]. Thus, targeting either macrophages and/or these tumor-promoting phenotypes may be instrumental to developing therapeutic opportunities to alleviate cancer burden (Figure 1).

### 8.1. Macrophage Depletion

While targeted depletion of macrophages as monotherapy in preclinical models of established solid tumors is beneficial in some instances [126,162,163], other studies have revealed that, when used as a single agent, macrophage targeting strategies are minimally effective at slowing growth in established tumors [164]. It is interesting to note that for some studies wherein monotherapy was advantageous, depletion strategies targeted resident macrophage populations in addition to TAMs [162]. Despite limited evidence of efficacy for single-agent therapies to deplete macrophages in solid tumors, a number of clinical trials investigating macrophage depleting agents have been undertaken [165] and have also shown limited efficacy. However, it is worth noting that macrophage depletion following tumor resection in a spontaneous mouse model of melanoma reduced recurrence and metastasis [166], thus, highlighting timing of therapy as a consideration to achieve maximal therapeutic success [164].

Early monocyte and macrophage depletion methods relied on the use of bisphosphonate-packed and clodronate-loaded liposomes [167,168,169]. Clodronate-mediated TAM ablation demonstrated the crucial role of macrophages in orchestrating early tumor formation and progression in a mouse model of mammary cancer [170]. Depleting TAMs using clodronate-loaded liposomes was also shown to augment the anti-tumor effects of the protein kinase inhibitor, sorafenib, on tumor angiogenesis, growth, and metastasis in a hepatocellular carcinoma model [171]. Moreover, other studies have shown that clodronate liposomes enhance the anti-tumor and anti-angiogenic effects of anti-VEGF antibodies in subcutaneous tumor models [172,173].

More recently, the use of trabectedin, an anti-cancer chemotherapy approved for the treatment of ovarian cancer and soft-tissue sarcomas, has been shown to induce apoptosis of monocytes and macrophages in tumor tissues via activation of caspase 8 and TRAIL-R2, a death receptor expressed exclusively on TAMs [174,175,176]. Currently, trabectedin is under investigation as a combination therapy with other anti-cancer drugs in phase I, II, and III clinical trials (reviewed by Lopez and colleagues [177]).

Inhibition of the CSF-1/CSF-1R signaling axis has been the most thoroughly investigated mechanism to deplete tumor-associated macrophage populations. A number of strategies have been developed to interfere with this macrophage-survival pathway including monoclonal antibodies targeting CSF-1 or CSF-1R, or small molecule CSF-1R inhibitors. Administration of two independent, chemically distinct, small molecule CSF-1R inhibitors, BLZ945 and PLX3397, were shown to deplete macrophages in healthy tissues [178,179]. Using tumor models, BLZ945 administration reduced macrophage populations in the liver and tumor while not affecting lung macrophages or circulating monocyte populations, thus, highlighting the differential susceptibility of this signaling cascade on the survival of macrophages of different origins [126]. In the same study, BLZ945-mediated TAM depletion was associated with increased CD8^+^ tumor infiltration and prevented tumor growth in a transgenic mouse model of cervical carcinoma [126]. Enhanced CD8^+^ T cell infiltration and improved response to chemotherapy was also observed in the spontaneous mammary carcinoma MMTV-PyMT mouse model following anti-CSF-1 antibody mediated macrophage depletion [145]. While investigating potential benefits of PLX3397-mediated macrophage depletion in a mouse model of PDAC, Zhu and colleagues reported that, although CSF-1R blockade increased CD8^+^ T cell infiltration, it also increased the expression of inhibitory checkpoint molecules on T cells [161]. In that study, the combination of CSF-1R blockade with anti-PD-1 or anti-CTLA-4 blocking antibody improved anti-tumor immunity in mice refractory to ICB [161]. A number of clinical trials investigating inhibition of the CSF-1/CSF-1R signaling axis in combination with other therapies, including ICB, are currently ongoing for advanced solid tumors (recently reviewed by Anfray and colleagues [6]).

Depletion of TAMs can also be achieved by targeting surface molecules, including CD52, scavenger receptor A (SR-A), folate receptor β (FR-β), and CD206 [180]. Preclinical studies have shown that the anti-CD52 antibody, alemtuzumab, depletes macrophages in a murine ovarian cancer model and significantly reduces tumor growth [181]. Its efficacy in the treatment of various cancers is currently being investigated in clinical trials (NCT00069238, NCT01361711, NCT01030900). Targeting SR-A using a small peptide SR-A ligand that outcompetes endogenous SR-A and, thus, recapitulates SR-A deficiency was shown to inhibit tumor cell migration and metastasis in mouse carcinoma models, making it a drug target worth investigating [182]. Another promising target, FR-β, is highly expressed on TAMs and is associated with advanced stage and lymph node metastasis [183,184]. A study using an experimental glioma model revealed that specific targeting of FR-β using a recombinant immunotoxin significantly depleted TAMs and reduced tumor growth [185]. To enhance therapeutic efficacy, many drugs are now designed as conjugates, which improves localization and increases the number of targets while also selecting for specific cell populations. CD206, the macrophage mannose receptor, can be targeted using a novel drug delivery system whereby a polysaccharide with high affinity for CD206 is conjugated with a bisphosphonate to target and trigger CD206 macrophage elimination. Using this treatment, researchers observed impaired angiogenesis and reduced tumor progression in a sarcoma mouse model [186]. More recently, researchers engineered oncolytic viruses expressing CD206 and FR-β-targeting bi- and tri-valent T cell engagers (BiTEs/TriTEs), leading to selective depletion of M2-like macrophages in the TME [187]. This powerful approach combines direct cancer cell cytotoxicity with depletion of tumor-promoting macrophages [187].

### 8.2. Recruitment

The majority of monocyte recruitment inhibitors are monoclonal antibodies or pharmacological targets designed to block the interaction between monocyte-chemotactic cytokines and chemokines and their receptors. There are currently several clinical trials testing the safety and efficacy of such treatments as single agents or in combination with other therapies [6].

Much of the research investigating monocyte/macrophage recruitment strategies as targets for anti-cancer therapy have centered around the CCL2/CCR2 axis given that CCR2 is overexpressed by many tumors and enhances monocyte recruitment into the TME [188]. Indeed, use of anti-CCL2 antibodies have been associated with reduced macrophage infiltration and tumor growth [188,189]. However, in the clinic, completion of a Phase II clinical trial using anti-CCL2 antibodies in castration-resistant prostate cancer found that treatment neither blocked the signaling axis nor did it reduce tumor burden [190]. Therapeutic approaches targeting CCR2 by pharmacological inhibitors, such as PF-136309, anti-CCR2, or small-interfering RNA (siRNA) knockdown, are still under investigation [180,191,192]. As mentioned previously, many macrophage targeting therapies will be most efficacious when used in conjunction with others rather than when used alone. A recent study revealed that CCR2 antagonism sensitized tumors to anti-PD-1 ICB therapy in a number of murine tumor and metastasis models. In these models, tumor regression was associated with increased mobilization and activation of CD8^+^ T cells [193]. While single-agent strategies targeting monocyte recruitment may be beneficial in alleviating pro-tumorigenic effects of recruited macrophage populations, resident macrophage populations will remain undisturbed and may thwart attempts of regaining tumor control.

### 8.3. Repolarization

The abundance of macrophages, their contribution to the TME, and their plasticity make them attractive targets not only for depletion but also for repolarization strategies. As discussed below, many experimental therapies have focused on repolarizing these tumor-promoting cells towards a tumor-suppressive phenotype to improve patient outcomes.

Engagement of toll-like receptors (TLR) with their cognate ligands is a well-described method of inducing macrophage repolarization. TLR agonists, including lipopolysaccharides, and lipoproteins, stimulate NFκB, activator protein 1 (AP-1), and interferon regulatory factor signaling pathways. This results in activation of M1-associated genes and production of pro-inflammatory cytokines, including TNFα, IL-12, and IL-6 [194]. Indeed, Poly:IC-mediated activation of TLR-3 in a murine model of colon cancer led to M1 macrophage repolarization and reduced tumor growth through activation of IFN-α/β signaling pathways [195]. Despite successes in pre-clinical models, use of systemic TLR agonists as anti-cancer therapy is hindered by toxicity [196]. However, intratumoral TLR agonist therapy [197] or use of drug delivery systems to directly target TLRs expressed by TAMs can mitigate toxicity. For example, administration of β-cyclodextrin nanoparticles loaded with TLR7/8 agonists to tumor-bearing mice led to effective drug delivery to TAMs with limited off-target effects. These TLR7/8 agonist-loaded nanoparticles were potent inducers of M2-to-M1 macrophage repolarization and sensitized mice to anti-PD-1 therapy in PD-1 refractory tumors [198]. In addition, activation of the cytosolic stimulator of interferon genes (STING) pathway, which results in type-I interferon expression, has also been associated with macrophage polarization [199] and potentiation of ICB responses [200] in pre-clinical models.

Efforts to deplete macrophages using small molecule inhibitors of the CSF-1/CSF-1R axis led to the unexpected finding that targeted populations could be instead repolarized towards anti-tumor phenotypes [161,178]. In a mouse model of GBM, Pyonteck and colleagues reported an absence of TAM depletion following CSF-1R inhibition. Instead, researchers found that TAMs were reprogrammed by glioma-secreted factors and exhibited anti-tumor gene signatures, which were also found to be associated with enhanced survival in patients with GBM [178]. Similarly, macrophage repolarization was observed following CSF-1R blockade in a mouse model of hepatocellular carcinoma wherein macrophage numbers were unaffected by PLX3397 treatment. In this model, delayed tumor growth was also associated with increased CD8^+^ T cell infiltration [201]. Lastly, in a model of PDAC, residual macrophages remaining following targeted CSF-1/CSF-1R depletion strategies displayed increased expression of anti-tumor immunity genes, reduced expression of immunosuppressive molecules, and enhanced antigen presentation capabilities, thus, contributing to improved CD8^+^ T cell responses directing anti-tumor efficacy [161].

The macrophage polarization process is tightly regulated through various signaling pathways, commonly those implicated in the inflammatory response. For example, phosphatidylinositol-3-kinase (PI3K)-γ, a key lipid kinase in macrophages, is a potent regulator of macrophage polarization that drives immunosuppressive transcriptional programs in macrophages and controls the switch between immune suppression and immune stimulation [202]. PI3Kγ has emerged as a target of interest over the past few years as researchers have started developing selective small molecule inhibitors to improve anti-cancer responses [203]. Blockade of PI3Kγ in a mouse model of PDAC abrogated macrophage-mediated suppression of adaptive immune responses and reprogrammed TAMs to instead promote CD8^+^ T cell-mediated tumor suppression, ultimately reducing invasion and metastasis [202]. Moreover, Gunderson and colleagues identified PI3Kγ-mediated regulation of Bruton’s tyrosine kinase (BTK) in macrophages as a key regulator of anti-tumor responses in a murine model of PDAC [204]. Specifically, administration of either a PI3Kγ inhibitor or BTK inhibitor (ibrutinib) to PDAC-bearing mice repolarized TAMs towards a Th1 phenotype and led to CD8^+^ T cell cytotoxicity and reduced tumor burden.

Targeting β-catenin/FOSL2/ARID5A signaling has been explored in cancer for its role in orchestrating recruitment of immune cells, including macrophages and T cells, to the TME [205,206]. This pathway has more recently been studied for its role in cancer cell-immune cell cross-talk in the TME. Indeed, pharmacological inhibition of β-catenin signaling results in repolarization of M2-like macrophages to M1-like macrophages, thereby reducing tumor growth and metastasis in a mouse model of lung cancer [207]. Furthermore, many of these inflammatory pathways converge to the activation of NF-κB, making it a valuable drug target. Embelin, a small-molecule inhibitor of the X-linked inhibitor of apoptosis protein, was shown to attenuate M2-like polarization of macrophages and reduce NF-κB-mediated release of pro-tumorigenic factors [208]. Modulation of the NF-κB pathway in macrophages to enhance anti-tumor responses was also achieved using mannosylated siRNA-delivering nanoparticles [209]. Moreover, use of a Listeria-based hepatocellular carcinoma vaccine was found to activate and repolarize TAMs from M2 to M1 through activation of the NF-κB pathway via TLR2 and MyD88 pathways, ultimately resulting in a tumor-suppressive TME [210].

Microbes are of great interest for researchers for their ability to re-educate macrophages. A great example is attenuated Listeria monocytogenes that have been shown to target pro-tumoral macrophages in the TME of ovarian cancer and repolarize these cells to an anti-tumor phenotype [211]. Furthermore, fungal products, such as β-glucan, which is a yeast-derived polysaccharide, have also been associated with macrophage repolarization. β-glucan treatment was shown to convert immunosuppressive macrophages in the TME to an anti-tumor phenotype, which was associated with slowed tumor growth and progression [212]. The use of β-glucan as an innate immune-modulating agent will be further discussed below.

Macrophages express a variety of membrane proteins, including scavenger receptors and co-stimulatory and inhibitory immune checkpoint molecules, some of which have been directly linked with macrophage activation and polarization. Indeed, antibody-mediated activation of CD40, a costimulatory protein found on APCs, was found to activate macrophages and enhance anti-tumor-responses [213]. CD40-mediated TAM repolarization was reported in both mouse and human PDAC [214,215] and in mouse models of GBM [216] and melanoma [217]. Additional work has revealed that treatment of macrophages with anti-PD-L1 resulted in the upregulation of multiple macrophage inflammatory pathways, including mTOR (mechanistic/mammalian target of rapamycin) and a reversal of pro-tumoral phenotypes [218]. MARCO, which becomes upregulated upon IL-37-induced M2 polarization, as explained above, is highly expressed on tumor-promoting macrophages and represents a therapeutic target for repolarization [219]. Antibody-mediated targeting of MARCO on TAMs was shown to repolarize macrophages; reduce tumor growth and metastasis; and enhance the effect of CTLA-4 ICB in murine models of mammary cancer, melanoma, and colon carcinoma [219]. As the IL-37/IL-37R signaling axis is upstream of MARCO-mediated tumor promotional programs of TAMs, it represents a therapeutic target for repolarization. Indeed, antibody-mediated blockade of MARCO and IL-37R, or CRISPR knockout of IL-37 in lung cancer cells, altered TAM phenotypes, resulting in reduced activity of regulatory T cells and enhanced cytotoxic function of both NK and T cells [74].

Research into the use of nanoparticles as a macrophage repolarization strategy has also begun to be explored in pre-clinical models. Whereas in vitro evidence revealed a Th1-skew in cytokine release from macrophages exposed to iron oxide nanoparticles, in vivo administration of iron oxide nanoparticles significantly inhibited subcutaneous adenocarcinoma tumor growth in mice and was associated with the increased presence of M1-polarized macrophages [220]. Similar in vivo macrophage repolarization responses were observed via direct targeting of CD206^+^ TAMs through the use of anti-CD206 antibody-conjugated iron oxide nanoparticles [221]. Moreover, to overcome issues of infiltration into solid tumors, direct targeting of the TME was achieved using pH-sensitive nanoparticles engineered to continuously release IL-12 in weakly acidic environments. This approach successfully repolarized macrophages in the TME to an anti-tumor phenotype with minimal toxicity [222]. Recent work has also investigated the therapeutic potential of engineering nanoparticles derived from CCR2-overexpressing monocytes loaded with iron oxide to facilitate ferroptosis, repolarize local macrophage populations, and reduce metastatic burden [223]. Repolarization of macrophages from M2 to M1 was also achieved using self-assembled dual-inhibitor-loaded nanoparticles to target CSF-1 and Src homology 2 domain-containing phosphatase 2 pathways, which resulted in anti-tumor efficacy in mammary and melanoma pre-clinical mouse models [224].

## 9. Exploiting Macrophages as Targeted Drug Delivery Systems

The exploitation of macrophages as drug delivery systems in cancer is now being explored [225]. Macrophages are abundant in many solid tumors and can account for up to 50% of the tumor mass [225,226], thus, making them a desirable platform for drug delivery to tumor cells. Moreover, macrophages exhibit tropism for hypoxic tumor regions [227], directly interact with tumor cells, and are phagocytic; thus, they can extensively internalize and hold considerable drug loadings [228] and are not constrained by leaky, discontinuous vasculature, which hinders extravasation and delivery of traditional therapeutics [229].

### 9.1. Chimeric Antigen Receptor Macrophage Therapy

Adoptive cell therapies based on modified dendritic cells, T cells, and NK cells have been successful anti-cancer treatments, especially in hematological malignancies [230]. With the exception of sipuleucel-T autologous cell immunotherapy for prostate cancer [231], efficacy of adoptive cell therapies has been limited in solid tumors [232,233]. Chimeric antigen receptor (CAR) macrophage (CAR-M) therapy is a very recent strategy being investigated to induce ECM degradation or direct tumor phagocytosis in order to target cancer growth and progression in solid tumors [234,235]. Zhang and colleagues engineered macrophages with CARs containing two regions: a variable region that binds to HER2 and an intracellular region made of CD147. HER2 binding and receptor activation leads to an increase in the expression of MMPs and subsequent degradation of the ECM [234]. CAR-Ms activating the CD147 pathway were shown to reduce ECM deposition, promote T-cell infiltration into the TME, and inhibit tumor growth in a 4T1 murine mammary cancer model [234]. Klichinsky and colleagues engineered CAR-Ms with the purpose of targeting mesothelin or HER2+cancer cells for antigen-specific phagocytosis [235]. In addition to enhancing tumor clearance and prolonging overall survival in xenograft mouse models, these CAR-Ms expressed pro-inflammatory cytokines, converted bystander M2 macrophages to an M1 phenotype, enhanced antigen presentation, promoted T cell infiltration, and were resistant to pro-tumorigenic effects of immunosuppressive cytokines in a humanized mouse model [235]. Hence, in addition to robust and specific anti-tumor activity, CAR-Ms may be important to help sculpt the TME, providing a beneficial anti-tumor effect [235].

### 9.2. Macrophage-Mediated Drug Delivery

Live cell-mediated drug delivery systems involve the use of host-derived monocytes and macrophages to function as ‘Trojan Horses’ to deliver drugs to a target tissue [228]. Once in the tissue, cell contents can be actively released via physical or chemical stimulations (i.e., temperature, light, magnetic field, or ultrasound) directed to the tumor site [227]. For example, a recent study described loading, delivery, and near-infrared laser release of doxorubicin from RAW264.7 cells in prostate tumors, resulting in slowed tumor growth when compared with macrophage-free doxorubicin treatment [236]. In another study, doxorubicin-loaded RAW264.7 cells were found to exhibit tropism towards mouse 4T1 mammary carcinoma cells and reduced tumor burden and metastasis [237]. Similarly, in other models, doxorubicin encapsulated liposome-carrying macrophages inhibited tumor growth in both subcutaneous and metastasis xenograft tumor models. In particular, drug-loaded macrophages accumulated in lungs and released more doxorubicin 24 h after administration compared with the amount of doxorubicin delivered to the lungs when macrophages were not used as carriers [238]. The use of monocytes and macrophages as drug delivery systems targeting experimental brain metastases of breast cancer [239] has also been explored. Macrophages loaded with nanoparticles effectively crossed the blood–brain barrier and homed to the brain, providing a novel drug-delivery method to target metastatic sites that are otherwise inaccessible [239]. Finally, development of a CD206 nanobody-based radioimmunotherapy system, which targets radionuclides to CD206^+^ TAMs, significantly slowed tumor growth in a mammary carcinoma model [240]. This method of CD206^+^ TAM-mediated radioimmunotherapy effectively enabled delivery of high-dose radiation to radioresistant hypoxic tumor regions resulting in control of tumor growth comparable to results achieved with administration of chemotherapy or anti-angiogenic therapy.

## 10. Macrophages and Trained Immunity: A Novel Therapeutic Target?

**Figure 2:**
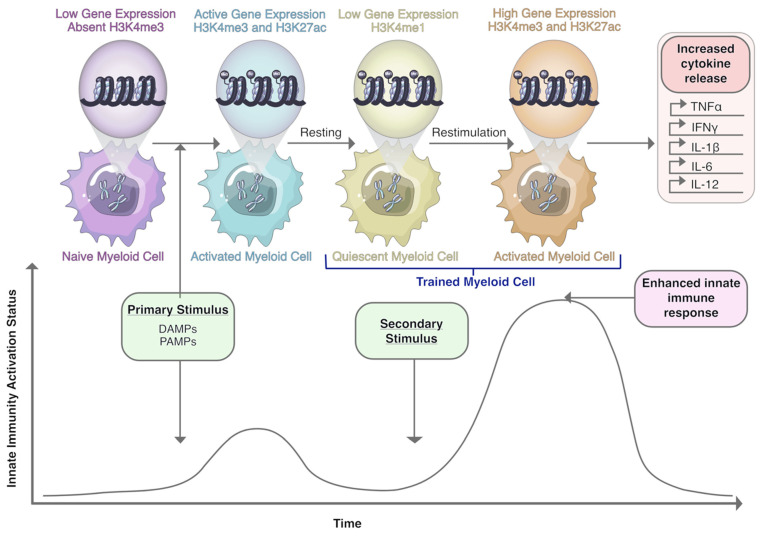
Acquisition of trained immunity in myeloid cells involves epigenetic reprogramming and results in enhanced inflammatory properties in innate immune cells. In response to primary exposure to DAMPs or PAMPs, the highly condensed chromatin of naïve myeloid cells relaxes following acquisition of methylation marks on histone proteins. Upon re-exposure to similar, but not necessarily identical, stimuli, the presence of these epigenetic modifications enables rapid transcription and expression of pro-inflammatory cytokines resulting in an enhanced innate immune response. H3K4me3, histone 3 lysine 4 trimethylation. H3K4me1, histone 3 lysine 4 monomethylation; H3K27ac, histone 3 lysine 27 acetylation; DAMPs, damage-associated molecular patterns; PAMPs, pathogen-associated molecular patterns; TNFα, tumor-necrosis factor-α; IFNγ, interferon-γ; IL-1β, interleukin-1β; IL-6, interleukin-6; IL-12, interleukin-12; me, methylation; ac, acetylation.

Trained immunity is a relatively new concept that describes the acquisition of immunological memory by innate immune cells through epigenetic reprogramming (i.e., methylation and acetylation of histones associated with pro-inflammatory genes) following exposure to DAMPs or PAMPs, such as those found on β-glucan or Bacillus Calmette–Guérin (BCG) [241,242]. Upon a subsequent exposure to a heterologous DAMP or PAMP, primed immune cells respond with a heightened release of Th1 pro-inflammatory molecules (e.g., IL-6, TNFα, and IL-1β; Figure 2) [241]. Differentiated myeloid cells, including monocytes, macrophages, microglia, and dendritic cells, as well as hematopoietic stem cell precursors, have all been shown to acquire trained immunity [242].

Evidence of trained immunity emerged after reporting heterologous beneficial effects of vaccines that could not be solely explained by adaptive immunological memory [243]. Early studies in mice revealed that BCG vaccination was protective against secondary infection by Candida albicans [244]. BCG is perhaps the most well-described inducer of trained immunity, and interestingly, intravesical BCG administration is the standard-of-care treatment for high risk non-muscle invasive bladder cancer (NMIBC) [245]. Forty to seventy percent of NMIBC patients treated with BCG suffer recurrences, and approximately 45% of these progress to muscle invasive disease within five years [246]. Although BCG has been used in the treatment of NMIBC for decades, our understanding of how it exerts its anti-tumor effects in patients is incomplete. In a study measuring urinary cytokines before and after BCG treatment, increases in proinflammatory cytokines, such as IL-6, IL-1Rα, and TNFα, were associated with lower risk of recurrence [247]. Moreover, monocytes isolated from patients treated with BCG responded with a threefold increase in proinflammatory cytokine production (IL-1β, IL-6, and TNFα) after ex vivo secondary stimulation with LPS [248], suggesting acquisition of trained immunity. Most recently, our group reported that trained immunity in circulating monocytes is associated with increased disease-free survival in bladder cancer patients treated with BCG [249].

Pre-clinical and clinical studies are now beginning to investigate whether other agents that induce trained immunity, for example β-glucan, have potential as anti-cancer therapies. Currently, there are several clinical trials investigating the use of β-glucan alone or in combination with other treatments to treat various cancer types (Table 1). A recent study by Kalafati and colleagues showed that trained immunity leads to transcriptomic and epigenetic rewiring of granulopoiesis, which in turn promotes an anti-tumor phenotype in neutrophils [250]. Whether acquisition of trained immunity promotes tumor suppressive phenotypes in macrophages is currently under investigation. Recently, Priem and colleagues developed a nanobiological therapy, MTP-HDL, that demonstrated remarkable anti-tumor efficacy by potentiating checkpoint inhibition in a mouse model of melanoma [251]. Trained immunity of bone marrow progenitors induced by MTP-HDL lowered TAM numbers and suppressed tumor growth. Moreover, the combination of MTP-HDL with either anti-PD-1, anti-CTLA-4, or both anti-PD-1 + anti-CTLA-4 ICB further enhanced the anti-tumor effect of MTP-HDL therapy [251]. Furthermore, the combination of training agents with ICB and therapies known to induce immunogenic cell death may be beneficial. Given that a hallmark of immunogenic cell death is release of DAMPs and PAMPs, the result would enhance anti-tumor responses in trained immune cells [252].

The epigenetic modifications required for trained immunity acquisition are similar to the epigenetic changes associated with differentiation of monocytes to macrophages [253,254]. When innate immune cells are trained and active, trimethylation of the fourth lysine in histone H3 (H3K4me3)—indicating open chromatin—is increased throughout the genome [253,254]. Monomethylation (H3K4me1) and acetylation of the 27th lysine (H3K27ac) also denote active regions and are known to occur in trained innate immune cells [253,254]. While the H3K4me3 and H3K4ac27 modifications are present within an active myeloid cell, the H3K4me1 mark persists and enables a faster response upon restimulation [253,254].

A recent study described the ability of monocyte-derived alveolar macrophages to retain ‘epigenetic legacy’ of their embryonic monocyte origin, resulting in protection against bacterial infection secondary to infection by influenza virus [255]. While this process is categorically different from the de novo epigenetic modifications described for trained immunity, it nonetheless underscores the idea that understanding epigenetic modifications in macrophage populations, whether newly recruited or tissue-resident cells, may be important to understanding how to harness the anti-tumor properties of these cells in the TME.

An important method of inducing this anti-tumor phenotype is through direct targeting of epigenetic regulators. There are various enzymes involved in regulating histone methylation and acetylation, including histone deacetylases (HDACs), which lead to transcriptional and phenotypic cell changes [256]. Guerriero and colleagues evaluated the effects of the class IIa HDAC inhibitor, TMP195, on TAM phenotypes and tumor burden in the macrophage-dependent MMTV-PyMT mouse model of mammary cancer [257]. Treatment with TMP195 increased the phagocytic and co-stimulatory capabilities of TAMs and led to reduced primary and metastatic tumor burden. Furthermore, durable anti-tumor responses were observed when TMP195 was combined with either checkpoint inhibitors or chemotherapy, thus, showcasing the potential of targeting epigenetic modifications in macrophages as a therapeutic strategy.

## 11. Conclusions

There is accumulating evidence that macrophages are key players in cancer evolution and, consequently, patient outcomes. Therefore, macrophages are obvious candidates for therapeutic intervention strategies. However, a deeper understanding of the biological role of these cells is needed to design more effective treatment options for patients with cancer. Phenotypic and transcriptomic differences among macrophages of different origins provide a rationale to consider macrophage ontology when studying macrophage-mediated tumor promotional pathways and potential targeting options. Despite significant advancements in development and implementation of immune therapy approaches targeting macrophages, many obstacles still remain and continue to be a barrier to successful patient treatment. When should treatment begin? Which treatments or combinations thereof will achieve long-term tumor control? Will there be significant toxicities associated with combination therapy? How can we improve treatment delivery methods to induce a potent but tumor-localized therapy response? With advancing research, these outstanding questions, and many others, will hopefully be resolved.

## Figures and Tables

**Table 1:** Summary of clinical trials investigating the use of β-glucan alone or in combination with other anti-cancer therapies ^1^.

NCT	Phase	Tumour Type	Therapy Name	Primary Outcome
**NCT00857025**	I	Lung Cancer	β-glucan MM-10-001	Safety, MTD, and toxicity
**NCT00682032**	N/A	Non-Small Cell Lung Cancer	β-glucan dietary supplement	Ability of β-glucan to prime neutrophils complement receptor 3, neutrophil cytotoxicity, and macrophage phenotype
**NCT04710290**	II and III	Metastatic Cancer	β-glucan dietary supplement	Total white blood cells, neutrophils, and lymphocytes at various times
**NCT00533364**	I and II	Breast Cancer	Soluble β-glucan	Safety of SBG in combination with standard antibody and chemotherapy treatment
**NCT04387682**	N/A	Squamous Cell Carcinoma of Oral Cavity	β-glucan dietary supplement	Recurrence-free survival or overall survival rate
**NCT00089258**	II	Neuroblastoma	β-glucan, isotretinoin, sargramostim, and 3F8 mAb	Disease response and efficacy
**NCT00087009**	1	Leukemia, Lymphoma, Lymphoproliferative Disorder	β-glucan and rituximab	MTD and safety
**NCT04513028**	N/A	Melanoma Stage III and IV	β-glucan dietary supplement and mAb 3F8	Lymphocyte cell surface expression markers
**NCT00037011**	I	Neuroblastoma	β-glucan and mAb 3F8	MTD and toxicity
**NCT00492167**	I	Neuroblastoma	β-glucan and mAb 3F8	Toxicity
**NCT00290407**	II	CLL, Small Lymphocytic Lymphoma	Rituximab and dietary supplement β-glucan	Clinical effect
**NCT01269385**	I and II	CLL	Alemtuzumab, rituximab, β-glucan (combination)	MTD
**NCT01829373**	I	Lung Cancer	Vaccine 1650-G and oral β-glucan	Immunological response to vaccine
**NCT00911560**	I and II	Neuroblastoma	Adjuvant OPT-821 in a vaccine containing two antigens (GD2L and GD3L) covalently linked to KLH and oral β-glucan	MTD and adjuvant effect of β-glucan.

^1^ MTD, maximum tolerated dose; mAB, monoclonal antibody; CLL, Chronic Lymphocytic Leukemia; KLH, Keyhole Limpet Hemocyanin; N/A, not applicable; SBG, soluble β-glucan.

## Data Availability

Not applicable.
